# Psychopathological Comorbidities and Clinical Variables in Patients With Medication Overuse Headache

**DOI:** 10.3389/fnhum.2020.571035

**Published:** 2020-11-27

**Authors:** Simone Migliore, Matteo Paolucci, Livia Quintiliani, Claudia Altamura, Sabrina Maffi, Giulia D’Aurizio, Giuseppe Curcio, Fabrizio Vernieri

**Affiliations:** ^1^Huntington and Rare Diseases Unit, Fondazione IRCCS Casa Sollievo della Sofferenza, San Giovanni Rotondo, Italy; ^2^UOS Cefalee e Neurosonologia, Neurology, University Campus Bio-Medico, Rome, Italy; ^3^Psychology Service, University Campus Bio-Medico, Rome, Italy; ^4^Department of Biotechnological and Applied Clinical Sciences, University of L’Aquila, L’Aquila, Italy

**Keywords:** emotion regulation, emotion recognition (ER), psychopatological profile, medication overuse headache (MOH), behavioral approach

## Abstract

The psychopathological profile of patients with medication overuse headache (MOH) appears to be particularly complex. To better define it, we evaluated their performance on a targeted psychological profile assessment. We designed a case-control study comparing MOH patients and matched healthy controls (HC). Headache frequency, drug consumption, HIT-6, and MIDAS scores were recorded. All participants filled in the following questionnaires: Beck Depression Inventory-II Edition (BDI-2), trait subtest of State-Trait Anxiety Inventory (STAI-Y), Difficulties in Emotion Regulation Scale (DERS), Barratt Impulsiveness Scale (BIS-11), Toronto Alexithymia Scale (TAS-20). The primary endpoint was to establish if MOH patients have an altered psychopathological profile. The secondary endpoint was to establish whether the worst profile correlates with the worsening of headache and disability measures. We enrolled 48 consecutive MOH patients and 48 HC. MOH patients showed greater difficulty in recognition/regulation of emotions (DERS, TAS-20), depression (BDI-2), anxiety (STAI-Y), and impulsiveness (BIS-11). We found a positive correlation among DERS, BDI-2, STAI-Y, and BIS scores and MIDAS and HIT-6 scores and among DERS and headache frequency and drug consumption. MOH patients showed a high rate of emotion regulation difficulties, depression, and anxiety, which may negatively affect their headaches. The ability to regulate/recognize emotions may play a central role in sustaining medication overuse.

## Introduction

The daily or almost daily frequent use of symptomatic drugs in patients with high frequency or chronic migraine, and less frequently with chronic tension-type headache, leads to the development of medication overuse headache (MOH).

The psychopathological profile of patients with MOH is very complex: together with mood and anxiety disorders, it can be observed as tending to obsessive-compulsive disorders and the occurrence of dependance-related behavior (Cupini et al., 2009; Radat and Lanteri-Minet, 2010; Lampl et al., 2016), and it has yet been suggested that a psychological profile assessment should be included in patients’ evaluation (Sarchielli et al., 2016). A negative prognostic value for psychiatric comorbidities has been suggested putting forward the hypothesis that these can represent a risk factor for the evolution of episodic into chronic headaches (Radat and Swendsen, 2005; Guidetti et al., 2010). Psychopathological disturbances are also seen as a potential predictor of relapse and poor response to treatment, and this can, in turn, complicate headache management facilitating MOH development (Cupini et al., 2009; Radat and Lanteri-Minet, 2010). Finally, some studies raised hypotheses about the potential comorbidity between psychiatric disorders and chronic headaches, but the presence of psychiatric disorders in MOH patients has been verified only in some of these (Buse et al., 2013; Sarchielli et al., 2016).

Our study aimed at evaluating the prevalence of psychopathological profiles in MOH patients through a comprehensive psychopathological battery to assess depressive symptoms and anxiety disorders, emotions’ recognition and elaboration, and impulsiveness’ level. We also investigated potential correlations between the psychopathological profile and some clinical variables (i.e., headache frequency, drug consumption, the impact of headaches on abilities of daily living). We expected MOH patients to show higher scores in psychopathological questionnaires compared with healthy controls (HC). Moreover, we hypothesized that psychopathological scores correlate with different clinical variables (i.e., monthly days of headache, medications taken per month, disease duration, and migraine-related functional disability).

## Materials and Methods

### Participants

We designed a case-control study comparing patients affected by MOH with HC, regarding possible differences in psychopathological profile. During the enrollment period (November 2015–May 2017), participation in the study was proposed to every outpatient with a MOH diagnosis that visited our Headache Center. MOH was diagnosed according to the International Classification of Headache Disorders, 3rd edition, beta version [Headache Classification Committee of the International Headache Society (IHS), 2013], based on clinical characteristics and the headache frequency resulting from personal headache daily diaries. Every other headache diagnosis was based on the ICHD criteria. Patients with suspected symptomatic headaches were investigated and excluded if needed.

Inclusion criteria for patients were age ≥18 years old, and fulfilling the ICHD 3rd edition, beta version criteria for MOH. Exclusion criteria were secondary headaches, and lack of inclusion criteria.

HC matched by age and gender were recruited among employees of the University Campus Bio-Medico. They all were free of medications at the moment of the assessment. Moreover, based on a clinical interview, we excluded those subjects who reported any known medical condition and neurological or psychiatric disease.

All patients had been under the care of our Headache Center for at least 3 months before the enrolment in the study and regularly completed their headache daily diary. We prescribed preventive therapy if patients were not taking it or a new one if they were. Patients were suggested not to overuse painkillers. Acetaminophen/paracetamol was allowed to treat the attacks. However, when patients were unable to refrain to take their usual symptomatic drugs, they were recommended to record in their diaries the number of triptans or NSAIDs or other analgesics they were forced to take. We reassessed patients after 3 months from the first visit. Mean headache frequency and symptomatic drug consumption in the previous 3 months were extracted from the diaries. If patients still overuse painkillers and the MOH diagnosis was confirmed, they had to start a bridge therapy protocol (Paolucci et al., 2017) for helping the withdrawal of symptomatic drugs. The protocol consisted of a 5-day iv infusion of saline solution NaCl 0.9% 250 ml with methylprednisolone 125 mg plus diazepam 10 mg, infused at 100 ml/h, and daily monitoring in a Day Hospital setting. Patients had not to take the overused symptomatic drug(s) and at the end of these 5 days received a new prophylactic therapy.

After informed consent was given, patients and controls were enrolled. At the moment of inclusion in the study, patients were asked to fill in a set of questionnaires to assess psychological profile, as described afterward. We also asked the patients to fill in Headache Impact Test (HIT-6; Bayliss et al., 2003) and Migraine Disability Assessment (MIDAS; Stewart et al., 2001) scores.

The primary endpoint was to establish if MOH patients have an altered psychopathological profile as compared to HC.

The secondary endpoint was to establish whether a worst psychopathological profile correlates with the worsening of headache impact and disability measures in MOH patients.

This study was designed following the ethical principles of the Declaration of Helsinki and all participants were asked to sign an informed consent. The study was approved by Campus Bio-Medico University Ethics Committee, approval number 44-18, and registered at AIFA (Italian Drug Agency) with number Eudract 2017-004606-18.

### Psychopathological Assessment

Patients, before starting bridge therapy protocol, and HC filled in a set of questionnaires to assess psychological profile, composed by:

1. Beck Depression Inventory-II Edition (BDI-2; Beck et al., 1996), a 21 multiple-choice questions self-report inventory to measure the severity of depression.
2. Trait subtest of State-Trait Anxiety Inventory (STAI-Y; Spielberger et al., 1983), a 20 multiple-choice items self-report questionnaire for measuring anxiety disorder.
3. Difficulties in Emotion Regulation Scale (DERS; Sighinolfi et al., 2010), a 36 items self-report questionnaire designed to measure multiple aspects of emotion dysregulation. The scale provides both a total score and scores on six subscales: non-acceptance of emotional responses (NONACCEPTANCE), difficulties engaging in goal-directed behavior (GOALS), impulse control difficulties (IMPULSE), lack of emotional awareness (AWARENESS), limited access to emotion regulation strategies (STRATEGIES), and lack of emotional clarity (CLARITY).
4. Barratt Impulsiveness Scale (BIS-11; Fossati et al., 2001) a 30 multiple-choice items self-report questionnaire for measure impulsiveness. The questionnaire provides a total score and 3 s-order factors, attention, motor, and non-planning impulsiveness.
5. Toronto Alexithymia Scale (TAS-20; Bressi et al., 1996) a 20 multiple-choice items self-report inventory for evaluating difficulties to identify and describe emotions. The scale provides a total score and three subscores, related to Difficulty Identifying Feelings (DIF), Difficulty Describing Feelings (DDF), and Externally and Oriented Thinking (EOT).

### Statistical Analysis

To better describe the psychopathological profile of patients and to highlight possible emotional dysregulation we decided to run a *post hoc* analysis partially based on previous data (Migliore et al., 2018). Chi-square test and Student’s *t*-test were run to assess the statistical difference between MOH patients and HC for sex and age distribution. Baseline headache measures were expressed as mean (SD) or median (IQR) depending on the variable distribution.

For the primary endpoint, to examine differences in experimental groups test performances, all dependent variables, obtained from psychopathological assessment scores (BDI-2, DERS, STAI-Y, BIS-11, TAS-20), were submitted to one-way ANOVA directly comparing the scores of two different groups (MOH vs. HC).

For the secondary endpoint, to highlight possible relationships between clinical variables (headache impact and disability measures: headache frequency, drug consumption HIT-6, MIDAS total score) and MOH psychopathological questionnaire performance, we initially performed bivariate correlation analysis. Taken into account that data did not respect the assumption of linearity and normality, we used Spearman’s correlation coefficient.

Alpha level was fixed to ≤ 0.05. All statistical analyses were performed using SPSS 25.

## Results

From November 2015 to May 2017 we enrolled 48 consecutive patients with MOH diagnosis (see Table 1 for clinical and demographic characteristics). We then enrolled 48 matched HC (Table 1).

**Table 1:** Demographic and clinical characteristics of the study sample.

	MOH patients (*n* = 48)	Healthy controls (*n* = 48)	*p*
Sex	F:38–M:10	F:37–M:11	*p* = 0.805
Age in years (mean ± SD)	47.7 ± 12.1	46.8 ± 10.71	*p* = 0.702
Disease duration in years (mean ± SD)	26.1 ± 15.1	-	
Monthly days of headache (mean ± SD)	24.1 ± 6.4	-	
Monthly drugs intake Median (min-max)	40 (12–315)	-	
MIDAS-total (mean ± SD)	88.8 ± 71.3		
MIDAS-A (mean ± SD)	61.2 ± 28.3	-	
MIDAS-B (mean ± SD)	8.2 ± 1.3		
HIT-6 (mean ± SD)	67.4 ± 5.6	-	

*Statistical comparisons refer to Chi-Square for the sex composition of the samples and Student’s t-test for mean age*.

Patients showed a mean of 24.1 days (± 6.4) of headache per month and a median of 40 symptomatic medications taken per month (IQR: 36; minimum 12 and maximum 315). The mean HIT-6 score was 67.4 (± 5.6), the mean of MIDAS total score was 88.8 (± 71.3), MIDAS-A score was 61.2 (± 28.3) and MIDAS-B score was 8.2 (± 1.3). We found no significant difference between men and women.

The group comparison showed that MOH patients and HC groups differed significantly in terms of total score of emotion regulation difficulties (DERS total score; *F*_(1,94)_ = 17.68; *p* < 0.00001; $\eta_{\text{p}}^{2}$ = 0.15), depression (BDI-2; *F*_(1,94)_ = 19.04; *p* < 0.0001; $\eta_{\text{p}}^{2}$ = 0.16), alexithymia (TAS-20 total score; *F*_(1,94)_ = 9.05; *p* = 0.003; $\eta_{\text{p}}^{2}$ = 0.08), anxiety (STAI-Y; *F*_(1,94)_ = 23.18; *p* < 0.00001; $\eta_{\text{p}}^{2}$ = 0.19). No difference was highlighted between groups in term of impulsiveness (BIS-11 total score).

When comparing the subscales of DERS, significant differences were observed in the Nonaccept score (*F*_(1,94)_ = 6.93; *p* = 0.01; $\eta_{\text{p}}^{2}$ = 0.06), Impulse score (*F*_(1,94)_ = 6.96; *p* = 0.01; $\eta_{\text{p}}^{2}$ = 0.06), Aware score (*F*_(1,94)_ = 6.55; *p* = 0.01; $\eta_{\text{p}}^{2}$ = 0.06), Strategies score (*F*_(1,94)_ = 16.28; *p* < 0.00001; $\eta_{\text{p}}^{2}$ = 0.14), and Clarity score (*F*_(1,94)_ = 7.31; *p* = 0.008; $\eta_{\text{p}}^{2}$ = 0.07); no differences emerged in Goal score. Regarding to BIS subscales, significant differences were observed in the Attention score (*F*_(1,94)_ = 7.7; *p* = 0.006; $\eta_{\text{p}}^{2}$ = 0.07); no differences highlighted in motor and no planning. Regarding to TAS-20 subscales, we observed statistical difference in DIF subscales (*F*_(1,94)_ = 16.47; *p* < 0.00001; $\eta_{\text{p}}^{2}$ = 0.15); no differences emerged in DDF and EOT subscores. The full details of the comparison results are shown in Table 2.

**Table 2:** Participants’ scores across the outcome variables.

Psychological questionnaire	MOH patients *Mean ± SD*	Healthy controls *Mean ± SD*	*p*
DERS (total score)	91.64 ± 26.03	73.31 ± 18.34	*p* < 0.001
DERS (subscore nonaccept)	16.65 ± 7.09	13.1 ± 5.96	*p* = 0.01
DERS (subscore goals)	14.87 ± 4.91	12.95 ± 5.16	*p* = 0.066
DERS (subscore impulse)	13.22 ± 5.85	10.54 ± 3.94	*p* = 0.01
DERS (subscore aware)	15.62 ± 4.38	13.31 ± 4.46	*p* = 0.01
DERS (subscore strategies)	19.62 ± 8.06	14 ± 5.31	*p* < 0.001
DERS (subscore clarity)	11.66 ± 5.21	9.29 ± 3.13	*p* = 0.008
TAS-20 (total score)	53.9 ± 14.09	46.23 ± 10.7	*p* = 0.003
TAS-20 (subscore DIF)	20.87 ± 7.09	15.54 ± 5.7	*p* < 0.001
TAS-20 (subscore DDF)	13.81 ± 4.7	13.39 ± 4.31	*p* = 0.65
TAS-20 (subscore EOT)	19.23 ± 5.35	17.29 ± 5.05	*p* = 0.072
BIS-11 (total score)	61.85 ± 8.12	58.72 ± 10.08	*p* = 0.098
BIS-11 (subscore attention)	16.68 ± 2.52	14.93 ± 3.53	*p* = 0.006
BIS-11 (subscore no planning)	22.54 ± 4.17	23.62 ± 5.65	*p* = 0.062
BIS-11 (subscore motor)	19.62 ± 3.8	20.16 ± 4.27	*p* = 0.514
BDI-2	18.8 ± 11.4	9.9 ± 8.4	*p* < 0.001
Trait subset of STAI-Y	48.92 ± 12.11	36.79 ± 12.55	*p* < 0.001

*Note: MOH, Medication Overuse Headache; DERS, Difficulties in Emotion Regulation Scale; NONACCEPT, non-acceptance of emotional responses; GOALS, difficulties engaging in goal-directed behavior; IMPULSE, impulse control difficulties; AWARE, lack of emotional awareness; STRATEGIES, limited access to emotion regulation strategies; CLARITY, lack of emotional clarity; TAS-20, Toronto Alexitimia Scale-20 item; DIF, Difficulty Identifying Feeling; DDF, difficulty describing feelings; EOT, externally oriented thinking; BIS, Barratt Impulsiveness Scale; BDI-2, Beck Depression Inventory 2; STAI-Y, State-Trait Anxiety Inventory*.

We found a significant correlation between basal HIT-6 score and depression (BDI-2; rs = 0.58; *p* < 0.0001), impulsivity both Attention (BIS-11; rs = 0.43; *p* ≤ 0.002) and total BIS-11 scores (rs = 0.33; *p* = 0.02), regulation of emotions (DERS nonaccept; rs = 0.4; *p* = 0.006; DERS goals; rs = 0.6; *p* < 0.0001; DERS strategies; rs = 0.53; *p* < 0.0001; DERS clarity rs = 0.45; *p* = 0.001; DERS total; rs = 0.58; *p* < 0.0001), trait anxiety (trait subtest of STAI-Y; rs = 0.6; *p* < 0.0001), and, finally, alexithymia (TAS-20 DIF; rs = 0.4; *p* = 0.006). Moreover, we found a significant correlation between headache frequency and regulation of emotions (DERS Aware; rs = 0.3; *p* = 0.04) and between number of medications and regulation of emotions (DERS Aware; rs = 0.3; *p* = 0.03). Finally, our analysis showed a significant correlation between basal total MIDAS Total score and depression (BDI-2; rs = 0.3; *p* = 0.04) and between MIDAS-B and trait anxiety (trait subtest of STAI-Y; rs = 0.28; *p* = 0.04). No correlation was found between psychopathological scores and disease duration.

## Discussion

Our study showed a significant difference in many psychopathological scales scores between MOH patients and HC subjects. Particularly, we demonstrated a high rate of depression, anxiety, and impulsiveness associated with a specific difficulty in recognizing and regulating emotions. Moreover, we found a positive correlation among psychopathological scales scores and both MIDAS and HIT-6 questionnaires, assessing the degree of migraine-related functional disability, showing that psychological comorbidities together with MOH negatively affect patients’ activities of daily living. Finally, we found a positive correlation between the DERS Aware subscore and some clinical variables, specifically headache frequency and the number of painkillers, but not with disease duration. This observation suggests that some of the psychological aspects evaluated are constitutional in patients with MOH and not the consequence of a long-standing pain condition. This relation between emotional dysregulation and pain intensity/analgesic consumption shows that the impairment in recognition/regulation of emotions producing an important dysfunctional behavior hugely impacts on disability from headache, regardless of the disease chronicity. To our knowledge, the present study is the first aiming to explore the emotions’ regulation abilities in a population of MOH patients. Besides, this is the first attempt also to explore the relationship between emotion regulation abilities and depression and anxiety in a MOH patients’ sample. Emotion regulation is the process of managing one’s emotions, but at the same time regards the “when” and the “how” individuals experience or express the emotions (Ciarrochi et al., 2001). Such a process involves both negative and positive emotions and when it works successfully can guarantee good mental health, as recently shown (Eftekhari et al., 2009). Difficulties in recognizing and regulating emotions have emerged in other neurological diseases, i.e Huntington Disease (Zarotti et al., 2018) and Multiple Sclerosis (Migliore et al., 2019). In the last years, also great attention has been paid to the nighttime involvement of emotional experience during dreaming, that correlated with volumetric and ultrastructural brain measures (e.g., De Gennaro et al., 2011). These findings suggested that difficulties in emotional skills (recognizing and regulating) may represent a precursor of more general cognitive impairment that could negatively impact daily life activities. Different reviews and meta-analysis (Di Tella and Castelli, 2016; Koechlin et al., 2018; Aaron et al., 2019) highlight as a recent growing body of researches is interested to evaluate emotion regulation’s role in different chronic pain (i.e., Complex Regional Pain Syndrome and Low Back Pain, Temporomandibular Disorders, Fibromyalgia, et cetera). These studies show significant emotion regulation difficulties in different types of chronic pain conditions. Emotion dysregulation may be an important risk factor in the development and maintenance of chronic pain and it is associated with many clinical (i.e., pain intensity) and psychological variables (anxiety and depression).

**Figure 1:**
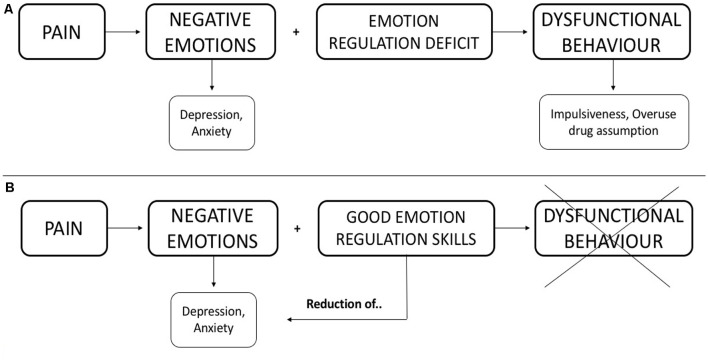
Hypothesized interaction among the discussed constructs. **(A)** We hypothesize that negative emotions associate with deficits in emotional regulation produce dysfunctional behavior. **(B)** We illustrate how good emotion regulation skills can limit dysfunctional behavior and, consequently, reduce negative emotions.

In MOH patients, it is possible to hypothesize that the chronic, almost daily, headache produces negative emotions. The MOH patient has difficulty coping with negative emotions (impairment in emotion regulation abilities) and this psychological feature can represent a specific condition that may generate dysfunctional behaviors (psychopathological symptoms). Figure 1A shows a schematization of the hypothesized interaction.

Furthermore, our study confirms the high rate of depression and anxiety symptoms in MOH patients, as highlighted by previous researches (Lampl et al., 2016; Sarchielli et al., 2016).

Several mechanisms have been proposed to explain the comorbidity of headache and psychopathological symptoms: (a) unidirectional or bidirectional causal models; (b) shared genetic factors; and (c) environmental risk factors. Overall, the lack of clear predictive relationships between psychopathological symptoms and headache raises the possibility either of a symmetrical causal link (i.e., each disorder would be a risk factor of the other), or of a common genetic or environmental risk factor (Radat and Swendsen, 2005). Indeed, the interactive effect between environmental risk factors and genetic factors could reasonably induce the development of both the considered diseases (Radat and Swendsen, 2005). On the contrary, the relationship between depression and migraine would appear to be bidirectional, i.e., each condition would increase the incidence of the other (Breslau et al., 2003).

We hypothesize that the altered ability, both to recognize and regulate emotions may play a central role in the behavior of patients with MOH. These altered behavioral abilities can contribute to the chronicization of head pain and to overuse of symptomatic drugs (as illustrated in Figure 1A), which is hardly treated only with a pharmacological approach.

The capability of recognizing emotions can allay a lot of negative emotions (moods) originating from headaches. In light of this, the evaluation of the psychopathological profile should be included in the general assessment of MOH patients. In this way, clinicians could plan an integrated treatment (both behavioral and pharmacological) to significantly improve MOH handling. Focusing early on the impairment of regulating emotions could have also a positive effect on anxiety-depressive symptoms and reduce dysfunctional behaviors (i.e., impulsiveness, overuse of drug assumption). We illustrated this hypothesis in Figure 1B.

Treating MOH patients, trying to reverse their compelling necessity to consume drugs, is a hard challenge for the headache specialist. Detoxification from MOH is a shared but not worldwide standardized practice used by headache units. Nevertheless, wash-out seems a useful protocol for treating medication overuse but only in the short term (Paolucci et al., 2017). There is a need for treating MOH comprehensively, including both a standardized pharmacological protocol and a psychological adequate approach. The battery of scales and questionnaires we used was useful in selecting patients with the highest degree of disability who might benefit from additional treatment approaches designed based on their individual profile of psychopathology.

The reason why some patients overuse acute treatments presenting MOH while others do not is not clearly understood. MOH might be related to some psychological states, such as fear and anticipatory anxiety of attacks, also defined as cephalalgophobia, The experience of recurrent severe pain may produce anticipatory anxiety for the forthcoming headaches and their consequence in terms of loss of daily activities which can be as dreadful as pain (Black et al., 2015). An alternative explanation relies on behavioral disorders, i.e., reward mechanism or compulsive disorder (Cupini et al., 2009). A drug-seeking behavior and the subsequent compulsive use of medications strongly complicate the drug withdrawal, which is the first step for treating MOH. So far, a direct link between compulsive behavior and medication overuse has not been established. Understanding the underlying mechanisms of those behaviors might improve the management of MOH.

The main limitation of our work is the lack of comparison with headaches or other pain conditions other than MOH. Future research is needed to consider patients with other headaches (i.e., migraine headache, tension headache, or cluster headache) and without MOH for evaluating the potential difference in the psychopathological profile and assess whether emotional dysregulation can be transversal to different forms of headache or specific to MOH. Moreover, since the sample size in the present analysis is rather small, prospective confirmation is needed in larger cohorts. Finally, it is necessary to investigate whether specific behavioral treatment (i.e., cognitive-behavior psychotherapy, biofeedback, and so on) can be effective in reducing psychopathological symptoms, improve quality of life, and improving the management of MOH patients.

## Data Availability Statement

The data that support the findings of this study are available from the corresponding author upon reasonable request.

## Ethics Statement

This study was designed following the ethical principles of the Declaration of Helsinki and all participants were asked to sign an informed consent. The study was approved by Campus Bio-Medico University Ethics Committee, approval number 44-18, and registered at AIFA (Italian Drug Agency) with number Eudract 2017-004606-18.

## Author Contributions

SiM was involved in the study design, clinical assessment, data collection, analysis, interpretation, and wrote the manuscript. MP was involved in the study design, clinical assessment, data collection, analysis, provided critical review and approval of the manuscript. LQ was involved in the analysis and interpretation of behavioral changes, provided critical review and approval of the manuscript. CA was involved in the study design, data collection, provided critical review and approval of the manuscript. SaM was involved in the interpretation of behavioral changes, provided critical review and approval of the manuscript. GD’A was involved in the study design, data analysis, provided critical review and approval of the manuscript. GC was involved in the study design, data analysis and interpretation, provided critical review and approval of the manuscript. FV was involved in the study design, data collection, data analysis and interpretation, provided critical review and approval of the manuscript. All authors contributed to the article and approved the submitted version.

## Conflict of Interest

The authors declare that the research was conducted in the absence of any commercial or financial relationships that could be construed as a potential conflict of interest.
